# Curtailing side effects in chemotherapy: a tale of PKCδ in cisplatin treatment

**DOI:** 10.18632/oncotarget.439

**Published:** 2012-01-31

**Authors:** Navjotsingh Pabla, Zheng Dong

**Affiliations:** ^1^ Department of Cellular Biology and Anatomy, Medical College of Georgia, Georgia Health Sciences University and Charlie Norwood Veterans Affairs Medical Center, Augusta, GA 30912; ^2^ Present address: Division of Biology, California Institute of Technology, Pasadena, CA 91125

**Keywords:** cisplatin, chemotherapy, side effect, protein kinase C δ

## Abstract

The efficacy of chemotherapy is often limited by side effects in normal tissues. This is exemplified by cisplatin, a widely used anti-cancer drug that may induce serious toxicity in normal tissues and organs including the kidneys. Decades of research have delineated multiple signaling pathways that lead to kidney cell injury and death during cisplatin treatment. However, the same signaling pathways may also be activated in cancer cells and be responsible for the chemotherapeutic effects of cisplatin in tumors and, as a result, blockade of these pathways is expected to reduce the side effects as well as the anti-cancer efficacy. Thus, to effectively curtail the side effects, it is imperative to elucidate and target the cell killing mechanisms that are specific to normal (and not cancer) tissues. Our recent work identified protein kinase C δ (PKCδ) as a new and critical mediator of cisplatin-induced kidney cell injury and death. Importantly, inhibition of PKCδ enhanced the chemotherapeutic effects of cisplatin in several tumor models while alleviating the side effect in kidneys, opening a new avenue for normal tissue protection during chemotherapy.

## INTRODUCTION

Cancer is the most devastating and intractable human disease. There are numerous ways to kill cancer cells, but most of them do not translate into cancer therapy because they kill normal cells and tissues as well. Thus selective and efficient targeting of cancer cells is the foremost goal in the development of anti-cancer therapeutics [1]. Most of the early anti-cancer therapies have some selectivity for cancer cells by inhibiting essential process like DNA replication and cell division in rapidly dividing cancer cells [1]. Recently developed anti-cancer drugs often have specific molecular targets that are deregulated in cancer cells [2]. The critical barrier in the use of early anti-cancer drugs is the development of resistance in cancer cells and the severe toxicity to normal tissues [3, 4]. It was believed that these impediments could be significantly reduced in the new generation of drugs that target specific molecular pathways in cancer tissues [2, 4]. However, it has been found that the new drugs often suffer from the same drawbacks [5].

Due to the side effect or toxicity in normal tissues, the therapeutic window of most anti-cancer agents is very narrow [6]. As a result, the anti-cancer drugs cannot be administered at the dosages where they can eradicate all the cancer cells. This is in part responsible for the selection of resistant cancer cells, ultimately leading to growth of tumors that are refractory to subsequent anti-cancer therapy [6]. Although, the significance of resistance to anti-cancer drugs has been widely recognized and extensively studied [7, 8], the research on the mechanisms responsible for the toxicity to normal tissues has generally lagged far behind. The identification of molecular targets that reduce toxicity to normal tissues without blocking the anti-cancer effects has the potential to significantly improve the efficacy of chemotherapy.

## CISPLATIN AND NEPHROTOXICITY

Cisplatin is one of the oldest, highly effective and most commonly used anti-cancer drugs [9-11]. Cisplatin is a platinum based inorganic compound that is believed to cross-link DNA, leading to inhibition of essential processes like DNA replication and transcription [11]. Higher rate of DNA replication in rapidly proliferative cancer cells make them particularly sensitive to cisplatin-induced DNA damage. Depending on the amount of DNA damage, cancer cells either repair/tolerate the DNA damage or undergo apoptosis in case of extensive damage [8]. Identification of resistant cells that have higher DNA repair or reduced DNA damage response/apoptosis have provided the basis that DNA damage is the major mechanism of cisplatin-induced tumor cell death [8, 12].

Along with its effectiveness in killing cancer cells, cisplatin has a wide range of side-effects in normal tissues, among which nephrotoxicity is most notable due to its potentially fatal nature [13, 14]. Cisplatin treatment can lead to severe kidney damage resulting in acute kidney injury, which has a very high rate of mortality [14]. Extensive hydration in patients can partially reduce the extent of kidney injury, however nephrotoxicity remains a main threat [13, 14]. Development of novel drugs derived from cisplatin like carboplatin and oxaloplatin have reduced toxicity, but these drugs are not as widely effective as cisplatin [13]. New strategies that reduce kidney injury during cisplatin treatment could have significant impact on the overall efficiency of chemotherapy.

Extensive studies have been conducted in the last decade to decipher the patho-physiological basis of cisplatin nephrotoxicity [13-17]. The major pathological feature of cisplatin-induced kidney injury is the cell death of renal tubular cells in the form of apoptosis and necrosis [13]. The specific sensitivity of tubular cells to cispatin is partly attributed to the fact that they accumulate many-fold higher amounts of cisplatin than other cells and tissues. Depending on the amount of cisplatin exposed, renal tubular cells undergo necrosis or apoptosis [18]. Along with renal cell death, an inflammatory component is also responsible for aggravating kidney injury [16, 19-22]. Injured kidney cells activate inflammatory processes, which further increase renal cell death leading to extensive kidney injury and acute renal failure [19]. Genetic and pharmacological strategies that inhibit both renal apoptotic pathways and inflammation in renal tissues provide significant protection during cisplatin treatment [13]. Other pharmacological/natural compounds that inhibit these processes indirectly also reduce cisplatin-induced kidney injury [13]. These studies have thus provided insight into the complex molecular mechanisms responsible for cisplatin nephrotoxicity. At the same time, it has been proposed that these strategies could have potential renoprotective effects during cisplatin-based chemotherapy.

However, the most critical issue that has not been addressed is the effect of these protective strategies on the anti-cancer efficacy of cisplatin. Indeed, some of the signaling pathways, for example the DNA damage response leading to p53 activation, observed in renal cells, also contribute to cisplatin mediated cancer cell death [23]. Identification of targets that can reduce renal toxicity without blocking the anti-cancer efficacy of cisplatin could have important clinical significance.

## IDENTIFICATION OF PKCΔ AS TARGET FOR RENOPROTECTION DURING CISPLATIN-BASED CHEMOTHERAPY

Protein kinase C (PKC) comprises of a family of highly conserved serine/threonine kinases that influence a wide range of cellular processes [24]. PKCδ is a member of the novel PKC sub-family, that are activated in a calcium-independent, but diacylglycerol-dependent manner [25, 26]. PKCδ is widely expressed in multiple tissues and has been implicated in a plethora of cellular processes including proliferation and apoptosis [25, 27, 28]. Knockout of PKCδ does not affect have overt effects on murine development, suggesting that PKCδ is not essential for normal mammalian development [29].

PKCδ is not only activated by cofactors, such as diacylglycerol and phorbol esters, but its activation is also dependent on post-translational modifications, especially phosphorylation [29]. Several tyrosine kinases, including growth factor receptors, Src family tyrosine kinases and c-Abl, have been implicated in PKCδ phosphorylation [29]. In addition PKCδ can be cleaved by caspases to generate a constitutively active catalytic fragment [25]. Numerous studies have thus implicated PKCδ activation as a pro-apoptotic mechanism during various stimuli, including treatment with anti-cancer agents [29]. Intriguingly, PKCδ can also function as an anti-apoptotic factor and confer resistance to anticancer drugs. Furthermore, PKCδ is pro-survival factor in several cancers [29]. Thus, depending on the treatment and cellular context, PKCδ may play contrasting roles in the decision of cell death or survival. The mechanisms underlying the dual roles of PKCδ remain unclear, but the localization, phosphorylation status and downstream substrates may be involved.

The lack of severe phenotype of PKCδ-deficient mice suggested that PKCδ might be dispensable for normal development and tissue maintenance [29]. However, PKCδ may play regulatory roles in cell stress and pathological conditions including treatment with anti-cancer drugs. Indeed our recent study [30] has identified a novel role of PKCδ during cisplatin-induced kidney injury. Genetic ablation and pharmacological inhibition of PKCδ provided significant renal protection during cisplatin treatment in *in vitro* cell cultures and *in vivo* murine models [30]. Mechanistically, PKCδ was shown to be activated in a Src-dependent manner leading to downstream activation of MAPK pathway.

**Figure 1:**
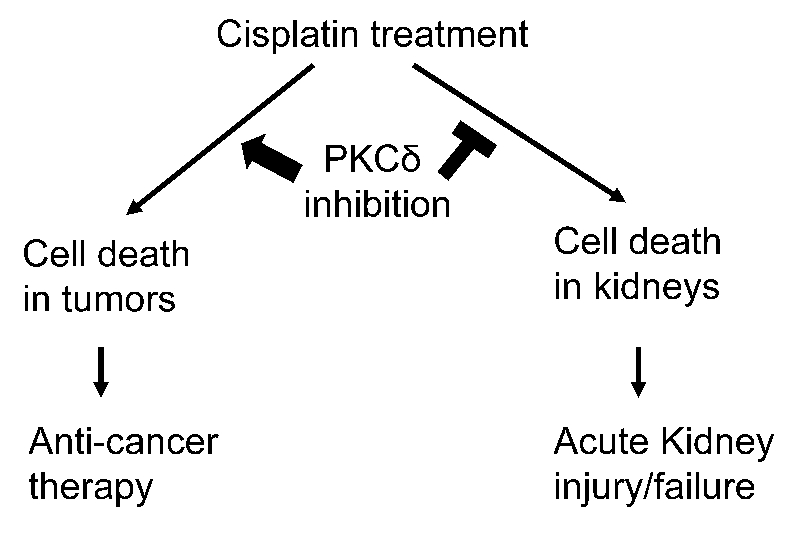
PKCδ inhibition enhances anti-cancer therapy while protecting kidneys during cisplatin treatment Cisplatin induces cell death in both cancer and kidney cells, resulting in chemotherapy in tumors and acute kidney injury and kidney failure. Genetic or pharmacologic inhibition of PKCδ enhances the chemotherapy effect of cisplatin in tumors and diminishes cisplatin-induced side effect in kidneys.

While our study suggested a renal protective strategy during cisplatin chemotherapy by targeting PKCδ, we were however concerned that PKCδ inhibition might attenuate the anti-cancer efficacy of cisplatin in tumors. To address this key issue, we conducted a series of *in vitro* and *in vivo* experiments to determine the effect of PKCδ inhibition on the anti-cancer efficacy of cisplatin. Intriguingly, we found that PKCδ inhibition did not reduce the effect of cisplatin in cancer cells and in some cases PKCδ inhibition actually increased the chemotherapy efficacy. Importantly, we developed new *in vivo* mouse models in which both the anti-cancer efficacy of cisplatin and its toxicity or side effects in kidneys could be directly monitored. These experiments demonstrated unambiguously that PKCδ inhibition reduced cisplatin toxicity in normal tissues without diminishing its anti-cancer effects. As a matter of fact, in ovarian and breast tumor models, PKCδ inhibitors enhanced the cancer therapy effect of cisplatin (Figure 1).

The observed different effects of PKCδ inhibitors in kidneys and tumors remain enigmatic. Earlier studies implicated a pro-apoptotic role of PKCδ in both normal and cancer cells [29], leading to the suggestion that PKCδ may function as a tumor suppressor. However, direct *in vivo* evidence for this theory is lacking and PKCδ-deficient mice do not display any propensity for carcinogenesis under normal conditions. In a recent study [31] the role of PKCδ in K-ras-dependent lung tumorigenesis was examined by using a mouse carcinogen model. Surprisingly, the incidence of urethane-induced lung tumors was significantly reduced in PKCδ-deficient mice compared with wild-type mice. PKCδ-KO tumors were smaller and showed significantly reduced proliferation. It is suggested that PKCδ may act as a tumor promoter downstream of oncogenic K-ras during lung carcinogenesis. Unexpectedly, these studies indicate that the function of PKCδ in tumor cells may depend on specific oncogenic context, as loss of PKCδ suppressed growth only in the cells that depend on oncogenic K-ras for proliferation and survival. Consistently, a pro-survival role of PKCδ has been documented in several cancer cell lines [29].

**Figure 2:**
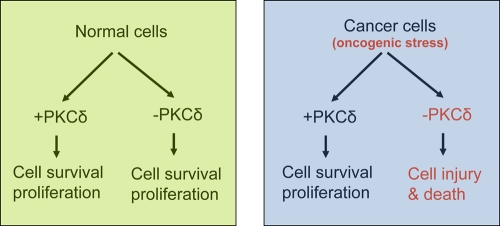
PKCδ in survival of normal versus cancer cells PKCδ is not required for the survival normal cells. However, it is required for the survival and proliferation of cancer cells with an oncogenic stress phenotype and, as a result, suppression of PKCδ leads to cell injury and death in tumors.

How can these seemingly contradictory findings be reconciled? We speculate that PKCδ may not have a critical pro-apoptotic or pro-survival role in normal mammalian development or physiological conditions. However, the cancer cells in some tumor types require PKCδ for survival and proliferation. Such scenario goes by the principle of ‘non-oncogenic addiction’[6] (Figure 1). According to this model, activation of oncogenes and rapid proliferation in cancer cells induce a stress phenotype that is particularly sensitive to cellular stress [6]. As a result, the cancer cells become dependent on certain pathways for survival, which are non-essential in normal cells and tissues. These non-oncogenic pathways are not important for initial cancer development, but are critical for the survival and proliferation of cancer cells in tumors. PKCδ seems to be a good candidate protein for ‘non-oncogenic addiction’ in cancer cells, which becomes essential for their survival under oncogenic stresses such as ras activation. If this is true, PKCδ could turn out to be an important target for anti-cancer therapy. Our study demonstrated a role of PKCδ in mediates the toxic effects of cisplatin in the kidneys and hence inhibition of PKCδ could reduce the toxicity and at the same time increase the anti-cancer efficacy of Cisplatin (Figure 2).

## CONCLUSIONS AND FUTURE DIRECTIONS

Our recent studies have shed light on a hitherto under-appreciated yet important aspect of cancer therapy. Since the side effect or toxicity to normal tissues is one of the key determinants in the success of anti-cancer drugs, identification of targets and development of normal tissue protective strategies could have significant implications in the treatment of cancer. Several strategies have been developed to selectively kill certain types of cancer cells, while simultaneously protecting normal cells [32-44]. By including normal tissue specific-protective agents in chemotherapy, these strategies may increase the therapeutic window. In line with this idea, PKCδ has now been demonstrated to be a critical component in nephrotoxicity during cisplatin treatment. The fact that PKCδ might be a “non-oncogenic addiction” factor for cancer cell survival provides an immense opportunity to increase the efficacy of chemotherapy by reducing the side effect in normal tissues and increasing the anti-cancer effect in tumors. Studies in the future should further elucidate the differential functions of PKCδ and other potential ‘non-oncogenic addiction’ genes in normal versus cancer tissues, opening new avenues to improve the efficacy of cancer therapy.
